# Laboratory testing and diagnostic coding for cytomegalovirus among privately insured infants in the United States: a retrospective study using administrative claims data

**DOI:** 10.1186/1471-2431-13-90

**Published:** 2013-06-07

**Authors:** Jessica Leung, Michael J Cannon, Scott D Grosse, Stephanie R Bialek

**Affiliations:** 1 National Center for Immunization and Respiratory Diseases, Centers for Disease Control and Prevention, 1600 Clifton Rd NE, MS A-34, Atlanta, GA, 30333, USA; 2 National Center for Birth Defects and Developmental Disabilities, Centers for Disease Control and Prevention, Atlanta, GA, USA

**Keywords:** Cytomegalovirus, CMV, Laboratory testing, Screening, Infants, Newborns, Administrative data, Medical claims, MarketScan®

## Abstract

**Background:**

Rates of laboratory testing and diagnostic practices for congenital CMV in the United States are unknown. We determined rates of CMV testing and diagnostic coding for CMV among insured infants in the United States using a national healthcare claims database.

**Methods:**

We analyzed medical claims from 2011 Truven Health MarketScan® Commercial databases for infants who were ≤30 days of age. We used ICD-9-CM codes to identify infants with CMV and CMV-associated conditions. We computed frequencies of infants with CPT codes for CMV testing.

**Results:**

A total of 368,266 infants met the study criteria. We identified 61 (0.02%) infants with a diagnostic code for CMV. Among the 368,266 infants, 229 (0.1%) infants had a code for CMV-specific testing, of which 43% had codes for CMV polymerase chain reaction (PCR) and/or CMV direct florescent antibody (DFA) testing, 44% for CMV serologic testing alone, and 13% for CMV serology and non-specific PCR and/or culture. Over 80% (187/229) with CMV testing had a code for ≥1 CMV-associated conditions. Although infrequently coded for, CMV testing was more common among infants with a code for a condition possibly associated with CMV than infants without these conditions (0.14% (187/ 136,857) vs. 0.02% (42/231,409)).

**Conclusions:**

The low rates of CMV testing among infants with symptoms suggestive of congenital CMV infection and the substantial proportion of infants tested with only serologic assays instead of PCR or viral culture suggests gaps in awareness and knowledge of congenital CMV and its diagnosis among healthcare providers. Although claims databases presumably do not capture all diagnosed CMV cases or CMV-specific testing, healthcare claims are a potential source for surveillance and monitoring practices of CMV-specific testing and diagnostic coding for CMV among infants.

## Background

Cytomegalovirus (CMV) is the most common congenital viral infection in the United States, affecting approximately 0.7% of all newborns, of which an estimated 90% will be asymptomatic at birth [1-4]. It is estimated that up to 0.2% of U.S. infants have signs and symptoms of congenital CMV infection at birth, based on retrospective analyses of infants diagnosed with congenital CMV [3]. Recognizing congenital CMV disease in the neonatal period can be challenging since manifestations such as microcephaly, chorioretinitis, and thrombocytopenia are not unique to CMV infection [5-7]. Consequently, few infants with even symptomatic congenital CMV are likely to receive a diagnosis in the absence of screening. Because 40-58% of surviving infants with congenital CMV infections that are symptomatic at birth will develop permanent sequelae [2], diagnosis of congenital CMV disease in the newborn period is important for identifying those with neurologic abnormalities who may benefit from treatment with antivirals as well as those who will need ongoing management of CMV-related sequelae such as hearing and vision loss.

Up to 12% of infants with congenital CMV infection that are asymptomatic at birth will develop CMV-related hearing loss later in childhood [2]. Routine newborn screening for congenital CMV infection is not currently recommended; rates of laboratory testing for congenital CMV infection and ascertainment of congenital CMV disease in the United States are unknown. Congenital CMV infection is confirmed by detection of CMV DNA by PCR or CMV by viral culture from urine, saliva, or blood [8]. In order to distinguish between congenital and postnatal infection, testing should be conducted within the first 2–3 weeks after birth [9] as infants may acquire CMV from infected cervical and vaginal secretions during delivery, breast milk feeding, or blood transfusions.

The objective of this study was to determine, in the absence of recommendations for routine screening for congenital CMV infection or disease, rates of coding for CMV diagnosis and testing among insured infants less than one month of age in the United States using a large national healthcare claims database.

## Methods

### Data source

We used healthcare claims data from the 2011 Truven Health MarketScan® Commercial Databases (Truven Health MarketScan Databases, Truven Health Analytics, Inc, Ann Arbor, MI) for approximately 50 million employees and their beneficiaries in the United States [10]. We used outpatient and inpatient claims data from the 2011 MarketScan Commercial Claims and Encounters databases, including information on demographics, health plan membership, International Classification of Diseases-9^th^ revision, Clinical Modification (ICD-9-CM) codes, and Current Procedural Terminology (CPT) codes. This study of de-identified data was determined not to require institutional review board review.

### Study definitions

Infants were 2011 enrollees ≤30 days of age. Since date of birth was not available in the MarketScan database, we used the date of the first claim with an ICD-9-CM code for live birth or ICD-9-CM V29 (observation and evaluation of newborns and infants) in 2011 to approximate the birth date as >90% claims with a newborn code occurred within 5 days of the first occurrence of the code.

Cases of congenital CMV were defined as infants ≤30 days of age with an ICD-9-CM code for congenital CMV infection (771.1) or CMV disease (078.5); we did not include laboratory confirmation of CMV infection because laboratory testing results were not available in the MarketScan database. CMV-associated conditions, identified by ICD-9-CM codes listed in Additional file 1: Table S1, were defined as 11 conditions included in previously published definitions of symptomatic congenital CMV [5-7] present in the newborn period (within the first month of birth). We defined “CMV-specific testing” as a claim with a CPT code for CMV IgG, IgM, direct florescent antibody testing (DFA), enzyme immunoassay (EIA) or polymerase chain reaction (PCR) and “non-specific viral or molecular testing” as a claim with a CPT code for non-specific viral culture or DNA molecular diagnostic testing (Additional file 1: Table S1). In order to characterize practices in laboratory testing for CMV, we looked at claims for all types of CMV-specific tests without restricting to laboratory tests used for diagnosing congenital CMV infection. We restricted our analyses for rates of claims for CMV-specific testing, and diagnostic coding for CMV and CMV-associated conditions to claims within 30 days of the newborn code.

Hearing loss is also a common symptom of CMV infection [2], although it may not be diagnosed in the newborn period. To examine diagnostic coding for hearing loss, we examined claims within the 1^st^ year of the newborn code. A diagnosis of hearing loss was defined as ≥3 medical encounters with a hearing loss code since the work-up for a diagnosis for hearing loss in infancy often requires >2 medical evaluations.

### Statistical analysis

To investigate the extent to which CMV testing among infants is captured by MarketScan data, we determined rates of CMV-specific testing among infants with ≥1 codes for other routinely recommended laboratory tests that are typically performed as part of standard infant care. These tests included phenylketonuria, hypothyroidism, galactosemia, and hemoglobinopathies (Additional file 1: Table S1).

We performed Pearson Chi Square or Fisher’s exact test to examine whether the presence of a code for CMV-specific testing was associated with a code for any of 11 CMV-associated conditions. The data were analyzed using SAS 9.2 statistical software (SAS Institute, Inc, Cary, NC).

## Results

### Infants with a diagnostic code for congenital CMV infection or CMV disease

Among the 368,266 infants ≤1 month of age, we identified 61 (0.02%) infants with a diagnostic code for congenital CMV infection or CMV disease. Of these, 63.9% (39/61) had an ICD-9-CM code for congenital CMV infection only, 27.9% (17/61) had an ICD-9-CM code for CMV disease only, and 8.2% (5/61) had ICD-9-CM codes for both congenital CMV infection and CMV disease. Seven (11.5%) had CMV-specific testing; none had a code for non-specific viral or molecular testing. Thirty-seven (61%) had an ICD-9-CM code for ≥1 CMV-associated conditions: 41% (25/61) had 1, and 20% (12/61) had 2–3 CMV-associated conditions. Low birth weight, jaundice, and thrombocytopenia were the most common CMV-associated conditions (Table 1).

**Table 1:** **Presence of a code for CMV-associated conditions**^**a **^**among all infants, infants with CMV-specific testing, infants with non-specific testing, and infants with a diagnostic code for CMV, MarketScan 2011**^**b**^

**CMV-associated condition**^**a**^	**All Infants**	**Infants with CMV-specific testing**^**c**^	**Infants with Non-specific testing**^**d**^	**Infants with an ICD-9-CM code for congenital CMV infection or CMV disease**
	**N=368,266**	**N=229**	**N=1,091**	**N=61**
	**# (%)**	**# (%)**	**# (%)**	**# (%)**
Chorioretinitis	13 (0.0)	0 (0.0)	0 (0.0)	0 (0.0)
Encephalitis	12 (0.0)	0 (0.0)	3 (0.3)	0 (0.0)
Hepatomegaly	45 (0.1)	5 (2.2)	1 (0.1)	0 (0.0)
Jaundice	110,997 (30.1)	54 (23.6)	361 (33.1)	11 (18.0)
Low Birth Weight	33,933 (9.2)	139 (60.7)	266 (24.4)	24 (39.3)
Microcephaly	115 (0.0)	5 (2.2)	7 (0.6)	3 (4.9)
Other Congenital Anomalies of Nervous System	988 (0.3)	24 (10.5)	28 (2.6)	4 (6.6)
Petechiae	1,650 (0.5)	1 (0.4)	2 (0.2)	1 (1.6)
Seizures	1,057 (0.3)	21 (9.2)	55 (5.0)	4 (6.6)
Splenomegaly	10 (0.0)	0 (0.0)	0 (0.0)	0 (0.0)
Thrombocytopenia	1,120 (0.3)	36 (15.7)	40 (3.7)	5 (8.2)
Any of the above 11 CMV-associated conditions	136,857 (37.2)	187 (81.7)	599 (54.9)	37 (60.7)
None of the above 11 CMV-associated conditions	231,409 (62.8)	42 (18.3)	492 (45.1)	24 (39.3)

^a^See Additional file 1: Table S1 for ICD-9-CM and CPT codes used to identify CMV-associated conditions.

^b^Infants are defined as enrollees ≤ 1 month of age with ≥1 medical claim within 30 days of a newborn code (defined as either a code for live birth, or observation and evaluation of newborns and infants; codes defined in Additional file 1: Table S1).

^c^CMV-specific testing includes CMV serologic testing (CMV IgG, CMV IgM, and CMV EIA), CMV DFA, and CMV PCR testing. (See Additional file 1: Table S1 for CPT codes).

^d^Non-specific testing includes viral culture, molecular diagnostics, or infectious agent detection. (See Additional file 1: Table S1 for CPT codes).

### Infants with CMV laboratory testing

Among the 368,266 infants, 229 (0.1%) had a code for CMV-specific testing, of which 7 infants (3.0%) had a diagnostic code for congenital CMV infection or CMV disease.

Among the 229 infants with CMV-specific testing, 98 (43%) had a code for CMV virologic testing (CMV PCR or DFA), 101 (44%) had a code for CMV serologic testing (CMV IgG, IgM, or EIA) alone, and 30 (13%) had a code for CMV serology and non-specific viral or molecular testing. Among the 101 with a code for CMV serologic testing alone, 37 (37%) had IgM testing alone, 16 (16%) had both IgG and IgM testing, 43 (43%) had IgG testing alone, and 5 (5%) had EIA testing alone. Most infants with a code for CMV-specific testing (187/229; 82%) had ≥1 codes for a potentially CMV-associated condition, while fewer of those with only non-specific viral or molecular testing (599/1091; 55%) did (Table 1). The most common CMV-associated conditions among infants with a claim for CMV-specific testing included low birth weight (61%), jaundice (24%), and thrombocytopenia (16%); 77% (177/229) of infants with CMV-specific testing had ≥1 codes for one of these three conditions. Although CMV-specific testing rates were statistically significantly higher in infants with hepatomegaly, jaundice, low birth weight, microcephaly, other congenital anomalies of nervous system, seizures, and thrombocytopenia than in infants without these conditions, rates of testing were low among infants with these conditions, ranging from 0-11%, with the highest rate of CMV testing occurring among those with hepatomegaly (Table 2). There were 542 (0.15%) infants with a diagnosis of hearing loss (≥3 visits for hearing loss within the 1^st^ year); 6 (1.1%) had CMV-specific testing in the first month of life.

**Table 2:** **CMV-specific testing**^**a **^**by presence or absence of a code for CMV-associated conditions among infants ≤1 month of age and infants with routine laboratory testing, MarketScan 2011**^**bc**^

**CMV-associated condition**^**c**^	**Infants** **N=368,266**			**Infants with Routine Laboratory Testing**^**d**^ **N=38,344**		
	**Total**	**Tested**	**P-value**	**Total**	**Tested**	**P-value**
	**#**	**# (%)**		**#**	**# (%)**	
Chorioretinitis						
No	368,253	229 (0.06)	1.00	38,340	129 (0.34)	1.00
Yes	13	0 (0.00)		4	0 (0.00)	
Encephalitis						
No	368,254	229 (0.06)	1.00	38,344	129 (0.34)	NA
Yes	12	0 (0.00)		0		
Hepatomegaly						
No	368,221	224 (0.06)	<0.01	38,336	126 (0.33)	<0.01
Yes	45	5 (11.11)		8	3 (37.50)	
Jaundice						
No	257,269	175 (0.07)	0.03	24,584	92 (0.37)	0.09
Yes	110,997	54 (0.05)		13,760	37 (0.27)	
Low Birth Weight						
No	334,333	90 (0.03)	<0.01	34,570	46 (0.13)	<0.01
Yes	33,933	139 (0.41)		3,774	83 (2.20)	
Microcephaly						
No	368,151	224 (0.06)	<0.01	38,328	127 (0.33)	<0.01
Yes	115	5 (4.35)		16	2 (12.50)	
Other Congenital Anomalies of Nervous System						
No	367,278	205 (0.06)	<0.01	38,206	116 (0.30)	<0.01
Yes	988	24 (2.43)		138	13 (9.42)	
Petechiae						
No	366,616	228 (0.06)	1.00	38,134	129 (0.34)	1.00
Yes	1,650	1 (0.06)		210	0 (0.00)	
Seizures						
No	367,209	208 (0.06)	<0.01	38,210	116 (0.30)	<0.01
Yes	1,057	21 (1.99)		134	13 (9.70)	
Splenomegaly						
No	368,256	229 (0.06)	1.00	38,343	129 (0.34)	1.00
Yes	10	0 (0.00)		1	0 (0.00)	
Thrombocytopenia						
No	367,146	193 (0.05)	<0.01	38,156	108 (0.28)	<0.01
Yes	1,120	36 (3.21)		188	21 (11.17)	
Any of the above 11 CMV-associated conditions						
No	231,409	42 (0.02)	<0.01	21,968	19 (0.09)	<0.01
Yes	136,857	187 (0.14)		16,376	110 (0.67)	

^a^ CMV-specific testing includes CMV serologic testing (CMV IgG, CMV IgM, and CMV EIA), CMV DFA, and CMV PCR testing. (See Additional file 1: Table S1 for CPT codes).

^b^Infants are defined as enrollees ≤ 1 month of age with ≥1 medical claim within 30 days of a newborn code (defined as either a code for live birth, or observation and evaluation of newborns and infants; codes defined in Additional file 1: Table S1).

^c^See Additional file 1: Table S1 for ICD-9-CM and CPT codes used to identify CMV-associated conditions.

^d^ Infants with ≥1 codes for other routinely recommended laboratory tests that are typically performed on infants as part of standard care. These tests included phenylketonuria, hypothyroidism, galactosemia, and hemoglobinopathies.

When we restricted the analysis to the 38,344 (10.4%) infants with ≥1 codes for other laboratory tests, a group which may have had more complete test claims data, our findings were similar. The overall rate of CMV-specific testing in this sub-group was 0.3% (129/38,344) with rates of testing ranging from 0-38% depending on presence of CMV-associated conditions, with the highest rate among those with hepatomegaly (Table 2).

## Discussions

This is the first study to examine the frequencies of diagnostic coding for congenital CMV and CMV laboratory testing among infants ≤1 month of age using healthcare claims data from a large insured population in the United States. We found that CMV-specific testing is rarely coded for infants and most CMV-specific testing appears to have been conducted for diagnostic purposes since 82% of those tested had a claim for at least one potential manifestation of congenital CMV disease. We found that 44% of CMV-specific testing conducted among infants ≤1 month of age appeared to be limited to serologic assays. Virologic testing is necessary for confirmation of congenital CMV infection because CMV IgG detected in newborns may reflect maternal antibody transmission and assays to detect CMV IgM antibodies lack sensitivity and specificity [7]. Although virologic testing was historically used as the gold-standard for diagnosing CMV, PCR testing for CMV is now widely accepted and the preferred method in some settings. While the low rates of CMV testing among infants with symptoms suggestive of congenital CMV infection and the high proportion of infants who appeared to have been tested only with serologic assays instead of PCR or viral culture may be explained in part by incomplete medical claims coding or bundling of services, these findings suggests gaps in awareness among healthcare providers of congenital CMV disease and its diagnostic evaluation.

We identified 0.02% of infants ≤ 1 month of age as having congenital CMV infection or CMV disease in a privately-insured population using healthcare claims data. That is a small percentage of the true rate of congenital CMV infection in the US population. The low rate of diagnosis of congenital CMV disease that we report likely reflects a combination of the insensivity of claims data in general to detect all diagnoses and tests included in medical records, and the low rate of clinical diagnosis of congenital CMV which could reflect low awareness among healthcare providers of congenital CMV disease as well as the non-specific nature of symptoms. Although healthcare claims databases are not sensitive enough to identify all symptomatic congenital CMV infections in the United States, they may be useful for surveillance to monitor trends in testing practices and diagnostic coding for congenital CMV disease in the absence of national surveillance for congenital CMV infection or disease.

An additional factor that might help account for the low frequency of diagnoses of congenital CMV in the present study is the selective nature of the MarketScan population, which represents individuals with employer-sponsored health insurance, about 55% of the US population in 2011 [11]. People with employer-sponsored insurance are less likely to be low-income or non-white than are uninsured or publicly-insured people [12]. Future work could examine the frequencies of coding for CMV diagnosis and CMV testing rates in the US population with publicly-financed health insurance.

There are a number of challenges to using claims data to investigate laboratory testing for congenital CMV infection. We were only able to identify infants with a diagnostic code for CMV infection, but were unable to confirm congenital CMV infection without access to laboratory test results or medical record review. The proportion of infants with CMV testing is likely underestimated as there is no specific billing code for CMV-specific viral culture. However, even if some of the claims for non-specific viral or molecular testing actually represented CMV-specific testing, the rate of CMV testing would still be quite low. It is unclear the extent to which CMV diagnostic testing was performed but not billed for with individual test codes; our finding that only 10% of infants had a code for one of the other routinely performed laboratory tests, suggests that there may be additional testing occurring that is not detectable because of bundling of charges. Many of the conditions associated with congenital CMV disease, such as jaundice, low birth weight, and hearing loss, are non-specific and have numerous potential underlying etiologies. It is difficult to definitively determine from medical claims data whether these conditions were due to congenital CMV infection but not recognized by providers as being CMV-related, coded to indicate an evaluation rather than diagnosis of CMV, or if an alternative etiology was identified. Some signs of symptomatic congenital CMV infection, such as petechiae and hepatosplenomegaly, are unlikely to be coded in claims data, even if recognized and recorded in the medical record. Finally, we were unable to validate the sensitivity and specificity of codes for CMV testing and diagnosis using medical claims. In the future, it would be useful to conduct a validation study of coding to enhance the interpretability of claims data for CMV testing and diagnostic coding.

## Conclusions

This study provides data on testing practices for congenital CMV infection among a sample of privately-insured infants in the United States. The substantial proportion of infants tested with only serologic assays instead of PCR or viral culture suggests gaps in knowledge of laboratory confirmation of CMV infection. Further investigation of the low prevalence of diagnosed symptomatic congenital CMV disease detected in this population merits further research to understand the true burden and spectrum of congenital CMV disease in this population, the extent to which symptomatic congenital CMV disease may go undiagnosed, and the validity of medical claims data for identifying this condition whether claims data could be an adequate source for surveillance to monitor trends in CMV testing and diagnostic coding in the United States. As the indications for and benefits of antiviral treatment for congenital CMV disease in early infancy are refined, increasing awareness and prompt ascertainment of congenital CMV is important to ensure identification of all infants who might benefit from early treatment.

## Abbreviations

CMV: Cytomegalovirus; ICD-9-CM: International Classification of Diseases, 9^th^ revision, Clinical Modification; CPT: Current Procedural Terminology; PCR: Polymerase chain reaction; IgG: Immunoglobulin G; IgM: Immunoglobulin M; DFA: Direct fluorescent antibody; EIA: Enzyme immunoassay.

## Competing interests

The authors declare that they have no competing interests.

## Authors’ contributions

JL conceptualized and designed the study; acquired, analyzed, and interpreted the data; drafted and critically revised the manuscript for important intellectual content; and approved the final manuscript as submitted. MJC conceptualized and designed the study; interpreted the data; reviewed and critically revised the manuscript for important intellectual content; and approved the final manuscript as submitted. SDG conceptualized and designed the study; interpreted the data; reviewed and critically revised the manuscript for important intellectual content; and approved the final manuscript as submitted. SRB conceptualized and designed the study; interpreted the data; reviewed and critically revised the manuscript for important intellectual content; and approved the final manuscript as submitted.

## Authors’ information

No additional information to add.

## Disclaimer

The findings and conclusions in this report are those of the authors and do not necessarily represent the official position of the Centers for Disease Control and Prevention, US Department of Health and Human Services.

## Pre-publication history

The pre-publication history for this paper can be accessed here:

http://www.biomedcentral.com/1471-2431/13/90/prepub

## Supplementary Material

Additional file 1: Table S1 List of International Classification of Diseases, 9th Revision. Clinical Modification (ICD-9-CM) and Current Procedural Terminology (CPT) codes. Click here for file
