# Supplementary material for: Laboratory testing and diagnostic coding for cytomegalovirus among privately insured infants in the United States: a retrospective study using administrative claims data
Source: BMC Pediatr. 2013 Jun 7;13:90. doi: 10.1186/1471-2431-13-90 (PMC3681590; doi:10.1186/1471-2431-13-90)
Supplement: Additional file 1: Table S1 — List of International Classification of Diseases, 9th Revision. Clinical Modification (ICD-9-CM) and Current Procedural Terminology (CPT) codes. [file 1471-2431-13-90-S1.doc]

**Laboratory Testing and Diagnostic Coding for Cytomegalovirus among Privately Insured Infants in the United States: a Retrospective Study using Administrative Claims Data**

**Appendix Table 1. List of International Classification of Diseases, 9th Revision. Clinical Modification (ICD-9-CM) and Current Procedural Terminology (CPT) codes**

| **ICD-9-CM/CPT Code(s)** | **Code Description** |
| --- | --- |
| **Laboratory Testing** | |
| CPT 86644-5 | CMV Antibody Testing |
| CPT 87271 | CMV Direct fluorescent antibody (DFA) Testing |
| CPT 87332 | CMV Enzyme immunoassay (EIA) |
| CPT 87495-87497 | CMV Infectious agent detection by nucleic acid (DNA or RNA) |
| CPT 87252, 87254 | Non-specific virus isolation by culture |
| CPT 83890-1, 83898, 83900-2, 83904-9, 83912, 87800-1 | Non-specific molecular diagnostics or infectious agent detection (DNA or RNA) |
| **Newborn Codes** | |
| ICD-9-CM V29 | Observation and evaluation of newborns and infants |
| ICD-9-CM V30-31, V33-4, V37, V39 | Live birth |
| **CMV and CMV-Associated Conditions** | |
| ICD-9-CM 771.1 | Congenital CMV Infection |
| ICD-9-CM 078.5 | CMV Disease |
| ICD-9-CM 363.0-3 | Chorioretinitis |
| ICD-9-CM 323 | Encephalitis |
| ICD-9-CM 389, 315.34, 388.2 | Hearing Loss (defined as ≥3 encounters with any one of these hearing loss codes in the first year) |
| ICD-9-CM 573.1, 789.1 | Hepatomegaly |
| ICD-9-CM 774 | Jaundice |
| ICD-9-CM V21.3, 764-5 (except 765.29) | Low Birth Weight |
| ICD-9-CM 742.1 | Microcephaly |
| ICD-9-CM 742 | Other Congenital Anomalies of the Nervous System |
| ICD-9-CM 772.6, 782.7 | Petechiae |
| ICD-9-CM 345, 779.0, 780.39 | Seizures |
| ICD-9-CM 789.2 | Splenomegaly |
| ICD-9-CM 776.1, 776.2, 287.3-287.5 | Thrombocytopenia |
| **Other routinely recommended procedures or laboratory tests for infants** | |
| CPT 84030 | Phenylketonuria (PKU) testing |
| CPT 84436, 84437, 84439, 84442-3, | Thyroid hormone testing |
| CPT 82775-6 | Galactose testing |
| CPT 83020-1 | Hemoglobin testing |
